# Trajectories of Pain in Low-Opioid and Opioid-Based Postoperative Analgesia in Older Patients—Perioperative Clinical Study

**DOI:** 10.3390/jcm14134416

**Published:** 2025-06-20

**Authors:** Urszula Kosciuczuk, Marcin Talalaj, Ewa Rynkiewicz-Szczepanska

**Affiliations:** Department of Anaesthesiology and Intensive Therapy, Medical University of Bialystok, Kilinskiego Street 1, 15-276 Bialystok, Poland; marcin.talalaj@wp.pl (M.T.); ewaryn@op.pl (E.R.-S.)

**Keywords:** analgesia, elderly, opioid, pain

## Abstract

**Background/Objectives**: The use of opioid drugs in the elderly population is characterized by an increased risk of sedation and respiratory depression, and in the immediate postoperative period, it is associated with a higher incidence of postoperative delirium. The dilemma of opioid use as an element of acute postoperative pain therapy is crucial in elderly patients. **Methods:** This study was conducted in 80 patients qualified for laparoscopic cholecystectomy under general combined anesthesia. Two methods of analgesia were performed—Low-Opioid Analgesia (LOA) and Opioid-Based Analgesia (OBA)—and pain intensity based on the Numerical Rating Scale (NRS) was assessed at 0–2, 2–6, 6–12, and 12–24 h after surgery. The mean NRS in LOA and OBA was compared in age categories. Pain trajectory in patients over 60 years old was compared between LOA and OBA. **Results**: The trajectory of analgesia presented a negative slope in LOA for patients over 60 years of age, with reductions in pain intensity of 33%, 25%, and 66%. In OBA, a positive slope trajectory was noted, and pain intensity was higher within 12–24 h after surgery than within 0–2 and 2–6 h. **Conclusions**: Opioid analgesia in patients over 60 years of age presented a better effect in the immediate postoperative period. Non-opioid analgesia is indicated for patients over 60 years old in the later postoperative period. The model of combined minimal opioid anesthesia and non-opioid postoperative analgesia presents a favorable therapeutic effect for patients over 60 years old.

## 1. Introduction

Epidemiological observations indicate that the population of patients over the age of 60 is increasing intensively, with a 21% increase over the last decade and an estimated global population of 1.5 billion by 2050 [1,2,3,4]. Consequently, more medical procedures—both surgical and anesthesiological—are performed among this group of patients. The epidemiological data document that more than 8 million surgeries per year have been performed on persons 65 years of age or older [1,5].

It has been shown that the risk of postoperative complications, including those directly threatening health and life, increases with age, and 25% of patients over 75 years of age experience postoperative complications [5,6]. One of these is uncontrolled postoperative pain and subsequent chronic pain [2,7,8].

Despite advanced surgical technology and anesthesiological methods, patients experience unrecognized and uncontrolled pain [2,5]. The perceived presence of postoperative pain is two times more common in adults over 60 years of age than in younger patients [3,9].

The predominant reasons explaining the underestimation of postoperative pain in patients over 60 are difficulties in reporting and articulating complaints, cognitive impairment, comorbidity in the advanced stage, limited regenerative and effective compensative mechanisms, and pharmacological polytherapy and related concerns about drug interactions [10,11,12]. Unrecognized and uncontrolled pain is particularly relevant to perioperative analgesia, especially in those experiencing severe pain requiring opioid drugs. Many publications have addressed the issue of using opioid-sparing analgesia perioperatively in elderly patients [3,4,5,6,7,8].

The use of opioid drugs in the elderly population is characterized by a greater sensitivity of neural tissue, which clinically results in an increased risk of sedation and respiratory depression, and in the immediate postoperative period, it is associated with a higher incidence of postoperative delirium [11,12]. It has been described that 6–14% of patients who are prescribed opioids after surgical discharge become persistent opioid users and continue their use for more than 3 months [13,14]. The worldwide trend of increased opioid drug prescribing in the immediate postoperative period and in the period of convalescence is also observed in the elderly population [14,15,16,17]. In addition, the use of this method of pharmacotherapy causes many changes in psychological functions and cognitive, behavioral, social, and immunological aspects [18,19,20,21,22].

Due to the well-recognized and well-described side effects and risks associated with the use of opioid drugs, methods of anesthesia and postoperative analgesia based on limiting opioid use have been described [23,24]. The classic method of anesthesia, associated with the use of opioid drugs in the induction phase of anesthesia and in the maintenance phase of anesthesia in fractionated doses or infusions, is Opioid-Based Anesthesia (OBA). Low-Opioid Anesthesia (LOA) involves the use of opioid drugs only in the induction phase of anesthesia, and intraoperative analgesia is provided through a multimodal analgesia method. The Opioid-free Anesthesia (OFA) rule advocates the complete avoidance of opioids during anesthesia and the use of multidirectional analgesic methods through the pharmacological synergism of analgesics and co-analgesics, regional analgesia, local anesthetics, and patient education [25,26,27,28,29,30].

General surgical operations, including cholecystectomies, are typical interventions in elderly people. The inhibition of gastrointestinal tract metabolism and peristalsis, as well as disorders of digestive secretion, cause gallbladder stones. Laparoscopic cholecystectomy, considered a minimally invasive surgical technique, causes an intermediate level of postoperative pain and, except for the use of opioid drugs during anesthesia (Opioid-Based Anesthesia), does not require the use of opioid drugs postoperatively; as a rule, non-opioid analgesia is sufficient [31,32,33,34].

Multimodal analgesia is the current trend in perioperative anesthesia care. This method is consistent with surgical expectations, providing an efficient process of recovery from anesthesia and convalescence. The key advantage is the reduction in opioid use, which possibly eliminates serious postoperative signs such as opioid hyperalgesia, decreased gastrointestinal motility, delirium, and impaired consciousness. For these reasons, multimodal analgesia is beneficial in elderly patients [25,26,27,28].

The aim of this study was to compare the analgesic trajectory of Low-Opioid Anesthesia (LOA) and Opioid-Based Anesthesia (OBA) for laparoscopic cholecystectomy surgery in a group of patients over 60 years of age based on the Numerical Rating Scale (NRS) for pain. In this study, we hypothesized that limiting the use of opioid drugs in LOA and a non-opioid postoperative analgesia regimen would provide a comparable analgesic profile to OBA.

## 2. Materials and Methods

Approval for this study was obtained from the Bioethics Committee of the Medical University of Bialystok No. R-I-002/105/2019. The exclusion criteria included a lack of consent to participate in the study, an ASA III and IV health status, a history of cancer and chronic pain, liver cirrhosis, epilepsy, addiction to drugs, alcohol, and psychoactive substances, and a history of postoperative nausea and vomiting, allergic reactions, or contraindications to analgesics. All patients were informed of the purpose of the intended study and how it was to be conducted, and they gave written consent. The inclusion criteria were as follows: patients with good clinical condition according to ASA 1 and ASA 2, who agreed to participate in the study, and who qualified for elective laparoscopic cholecystectomy.

This study is a non-randomized cohort study. Patients qualified for laparoscopic cholecystectomy were assigned to an anesthesia group based on the following order: the study group was recruited first, followed by the control group. The study included 80 adult patients undergoing laparoscopic cholecystectomy under combined general anesthesia with intravenous induction of anesthesia (propofol, 2 mg kg^−1^i.v, Fresenius) and volatile maintenance of anesthesia (sevoflurane, Baxter) with the use of cisatracurium (0.15 mg kg^−1^i.v, Kalcex) as a skeletal muscle relaxant.

Non-invasive monitoring of vital functions was performed: electrocardiography, systolic blood pressure, diastolic blood pressure, mean blood pressure, and pulsoxymetry. Volatile anesthesia was controlled according to the minimal alveolar concentration value, with a targeted level of MAC for sevolurane of 0.8–1.0, using the low-flow method. Muscle relaxation was controlled by using the accelerometrical method.

### Analgesic Scheme in Study and Control Group

1. Study group: forty patients with Low-Opioid Analgesia (LOA).

Twenty minutes before the induction of anesthesia, these patients received 1 g of intravenous paracetamol (Fresenius Kabi, Hamburg, Germany), a slow infusion of magnesium sulfate (Inj. Magnesii Sulfurici, Polpharma, Gdansk, Poland) at a dose of 2 g, and a slow infusion of lidocaine (Lidocainum Hydrochloridum WZF 1%, Polfa, Warsaw, Poland) at a dose of 100 mg. Immediately before the induction of anesthesia, ketamine (Ketlar 50, Pfizer, Kent, UK) was administered in a slow bolus of 50 mg, with fentanyl at 0.1 mg during induction (Fentanyl WZF, Polpharma, Warsaw, Poland). During the maintenance phase of anesthesia, there was continuous infusion of lidocaine (2 mg mL^−1^ solution), at a dose of 1.5–3.0 mg kg^−1^ h^−1^, and ketamine (1 mg mL^−1^ solution), at a dose of 0.125–0.25 mg kg^−1^ h^−1^, using the Braun Perfuzor Compact (Melsungen, Germany). In addition, 30 min before awakening, 2 g of metamizole (Pyralgin, Polpharma, Gdansk, Poland) was administered intravenously, while the ketamine infusion was stopped 10 min before the end of surgery, and the lidocaine infusion was terminated at the end of anesthesia.

2. Control group: forty patients who received general anesthesia with the opioid drug fentanyl (0.1 mg i.v) in the induction and maintenance phases of anesthesia based on the patient’s clinical condition (clinical hemodynamic signs, autonomic signs), with 2 g of metamizole administered intravenously 30 min before awakening, i.e., employing Opioid-Based Analgesia (OBA).

3. Postoperative analgesia

A standardized postoperative analgesic scheme was used for each patient and included the administration of 1 g of paracetamol and 1 g of metamizole intravenously with a 6 h interval between doses. Paracetamol was used as the basic first-line drug, and doses of metamizole were used as the second-line drug. Depending on the degree of pain control, patients could opt out of the planned doses of analgesics.

The severity of the pain was assessed at successive time intervals after surgery, including the first 2 h after surgery and between 2 and 6, 6 and 12, and 12 and 24 h after surgery. For this purpose, a numerical scale was used, according to which the number “0” indicates the absence of pain and the number “10” expresses the most severe level of pain imaginable.

Data are presented as the median, minimum–maximum range, and 25–75th percentile range (IQR), or as counts (n) with proportions (%). The Shapiro–Wilk test was used to check the normality of the distribution of the variables. In further analysis, non-parametric tests were used to present correlations between variables and between groups. The Mann–Whitney U test was used to compare two independent groups, and the Wilcoxon paired t test was used to compare variables. All calculations were performed using Statistica 14.0.0 (TIBCO Software Inc, Cracow, Poland.), and *p* < 0.05 was used as the level of significance.

This was a pilot prospective study; therefore, the sample size was determined as 40 subjects in the experimental group. The sample size was estimated based on the expected NRS differences, with an alpha value of 0.05 and power at 0.8, requiring a minimum group size of 21.

## 3. Results

The study group included 39 patients, while the control group included 37 patients. The reasons for excluding patients from the study were discrepancies in the planned postoperative analgesic treatment regimen and the administration of unplanned drugs. The characteristic data of the study and control groups are shown in Table 1.

The LOA group consisted of 11 patients with ASA 1 and 28 patients with ASA 2. In the OA group, there were 11 patients with ASA 1 and 26 patients with ASA 2. Hypertension arterialis, diabetes mellitus, nicotinism, asthma, and arrhythmias were the most common clinical conditions in ASA 2. The groups did not differ in anthropometric parameters: age, weight, BSA, and BMI. There were no events of surgical conversion to laparotomy. The groups did not differ in parameters of surgery and anesthesia.

The analysis revealed that, in the LOA group, a mean NRS of 0 was recorded in four patients, accounting for 10.25% of the group. Pain during the first postoperative day was reported by 89.75% of patients. No mean NRS of 0 was recorded in the OBA group, and all of these patients experienced pain of varying intensity.

The mean pain intensity on the first postoperative day, according to the Numerical Rating Scale (NRS), was not significantly different in patients under 40 years of age: the median, IQR, and min–max ranges were 2.37, 2.12–2.87, and 2–3.25, respectively, compared to 4.0, 3–8, and 2–8 in the control group (*p* = 0.13). In the group of patients over 60 years of age, there were no statistically significant differences in the NRS score, at 1.62, 1.5–2.25, and 0–7, respectively, compared to 2.0, 2–3.5, and 1–5 in the control group (*p* = 0.102). In contrast, the mean pain intensity was significantly lower among patients 41–60 years of age in the LOA group compared to the OBA group, recorded as 2.25, 1–3, and 0–4, respectively, compared to 4.0, 2–5, and 1–7 in the control group (*p* = 0.003).

**Figure 1:**
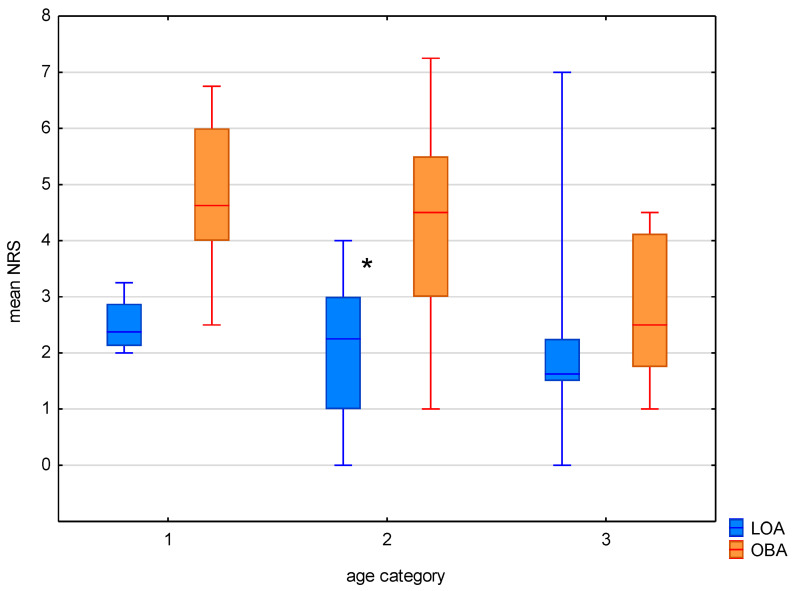
Mean pain in NRS in age categories between LOA and OBA. Median, IQR, min–max ranges are presented. *-statistical significance with *p* < 0.05.

The mean pain intensity in patients over 60 years old in the LOA group on the first postoperative day was 0.49 NRS points lower compared to the group aged 18–40, and it was 0.07 NRS points higher compared to the patients aged 41–60. In the OBA group, the mean pain intensity was the lowest in the group of patients over 60 years of age, at 2.17 NRS points lower than patients under 40 years of age and 0.88 NRS points lower than patients aged 41–60 (Figure 1).

**Figure 2:**
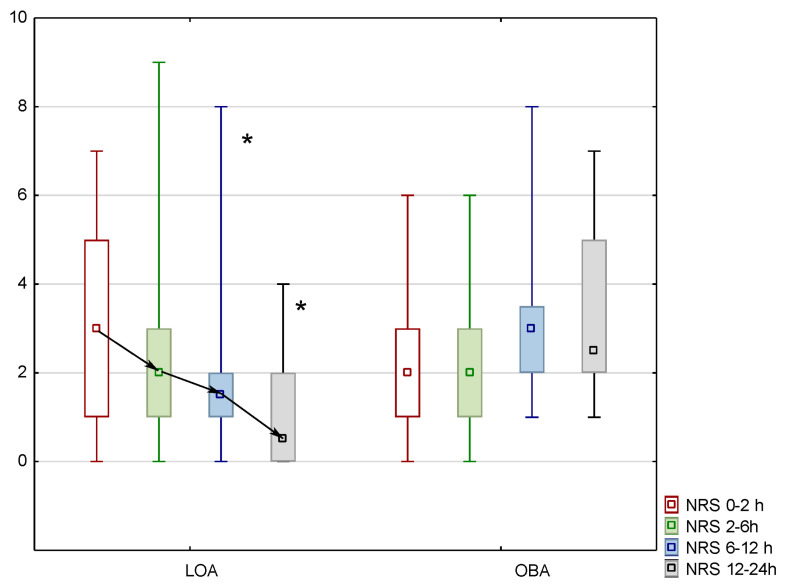
Pain intensity in NRS in patients over 60 years old in LOA and OBA. Negative slope inLOA. Median, IQR, min–max ranges are presented. *—statistical significance with *p* < 0.05.

The pain intensity in the group of patients over 60 years old in the OBA group was lower in the immediate postoperative period (0–2 h); the median, IQR, and min–max range were 2, 1–3, and 0–6, respectively, while in the LOA group, they were 3, 1–5, and 0–7. In the LOA group, the pain intensity was significantly lower in the postoperative period of 6–12 h (median, IQR, and min–max range of 1.5, 1–2, and 0–8, respectively, in the study group and 3, 2–3.5, and 1–8, respectively, in the control group (*p* = 0.012)) and 12–24 h (median, IQR, and min–max range of 0.5, 2–2, and 0–4, respectively, in the study group and 2.5, 2–5, and 1–7, respectively, in the control group (*p* = 0.0009)). The trajectories of analgesia at 24 h after surgery presented a negative slope in the LOA group for patients over 60 years old. There was a reduction in the pain intensity of 1 NRS point, 0.5 NRS points, and 1 NRS point, representing 33%, 25%, and 66%, respectively, related to the baseline values (Figure 2).

The mean values of fentanyl used in the OBA group did not differ significantly across age categories and were 0.23 mg for category 1, 0.28 mg for category 2, and 0.26 mg for category 3 (*p* < 0.05).

In the general study group, only 20% of patients used a constant prescription of paracetamol at 1 g every 6 h, totaling a dose of 4 g per day. None of the patients used the maximum dose of metamizole—4 g. In the group of patients over 60 years of age anesthetized via the LOA method, the need for non-opioid drugs was lower than in the OBA group. The average daily dose of paracetamol and metamizole was 3 g and 2 g in the LOA group vs. 3.5 g and 3 g in the OBA group, respectively.

## 4. Discussion

In our study, postoperative pain on the first day occurred in nearly 90% of the patients in the study group; in addition, all patients in the control group experienced pain of varying intensity. Only in the group of patients over 40 years of age in the LOA group was a mean NRS score of 0 recorded, which accounted for 10% of patients. In the group of patients over 60 years of age with LOA, the pain intensity was significantly lower at 6–12 and 12–24 h after surgery than in the OBA group.

Acute postoperative pain affects 80% of patients, with 86% experiencing moderate or severe pain, and the lack of effective analgesia results in chronic postoperative pain and affects up to 50% of patients [3,7,35,36].

After general surgery, postoperative pain persisted after 3 months in 26% in patients over 60 years old, and it was most commonly associated with orthopedic surgery, at 48%. After 6 months, there was a reduction to 14% after general surgery and 31% after orthopedic procedures. Persistent pain at 3 months was related to urological procedures in 27% and thoracic procedures in about 30%, and after 6 months, pain affected 14 and 20% of these patients, respectively. Due to long-term pain, 42% of postoperative patients underwent analgesic therapy 3 months after surgery, with half of them continuing after 6 months. In addition, long-term analgesic therapy was associated with the occurrence of mood disorders in 40% and social and occupational exclusion in 15% [1].

Age and gender were identified as the two dominant factors in pain perception. A study on the general population of patients undergoing operations reported that the average intensity of postoperative pain decreased by 0.042 NRS points per hour on the first day and was 0.27 NRS points lower in males compared to females. With regard to age, there was a reduction of 0.053 points in the NRS score per year of age. A reduction of 0.056 NRS points per 1 year of age for males and 0.052 NRS points per 1 year of age for females was observed. The clinical findings indicate that female sex is a risk factor for the occurrence of long-term pain and that women experience more severe pain as their age increases compared to men. The pain intensity significantly decreased on postoperative day 1, and the mean NRS score was 0.046 NRS points lower per hour in the 21–39-year-old age group, 0.042 NRS points lower per hour in the 40–64-year-old age group, and 0.041 NRS points lower per hour among patients over 65 years of age. These results indicate longer persistence of pain in older patients [7].

In this study, the OBA model met the principles of a positive slope in all age categories. In patients over 60 years of age, the LOA method met the conditions of a negative slope with favorable postoperative analgesia. Negative slopes mean that pain decreases with time, a flat slope is characteristic of stable pain perception, and positive slopes mean that pain increases with time. The positive slope model has been shown to predispose chronic pain. These models are used to depict the dynamics of pain perception in specific surgeries. Nervous surgery and digestive surgery tend to present negative slopes of0.054 NRS points per hour and 0.104 NRS points per hour, respectively. In contrast, the positive slope trajectory is typical for musculoskeletal surgery at + 0.02 NRS points per hour [7]. In addition, the trajectories have clinical applications in planning the duration and advancement of analgesic therapy. Pain therapy with a positive slope trajectory requires systemic opioid drugs in the early postoperative period and regional anesthesia with epidural administration of opioids and local anesthetics [7,35].

Operative risk factors for chronic postoperative pain have been identified: type of surgery, surgical technique, type of anesthesia, and severe motor-related postoperative pain on the first postoperative day. Surprisingly, the correlation of comorbidities, especially cardiovascular disorders, with prolonged postoperative pain has not been described [36,37,38,39,40].

A minimally invasive surgical technique was associated with a reduction in pain after 3 and 6 months, at 51 and 47%, respectively, while an increase was noted for open surgery, at 48% after 3 months and 52% after 6 months. It was also reported that pain was experienced similarly at 3 and 6 months after general anesthesia, at about 92%, compared to regional anesthesia (9.8 and 7.4%, respectively). After operations performed with peripheral nerve blocks, chronic pain was reported after 3 and 6 months in about 47% of subjects. The average pain intensity at rest within 24 h after surgery was 2 on the NRS and did not affect pain sensation reported after 3 and 6 months. However, the mean intensity of pain during movement within 24 h after general surgery was 4.2 on the NRS and showed a correlation with persistent pain [1]. In our study, we performed an analgesic evaluation of acute pain on postoperative day 1, with a special focus on patients over 60 years of age. At the final clinical follow-up (12–24 h), the maximum pain intensity values were four NRS points in the LOA group and seven NRS points in the control group.

One study reported that 72% patients after laparoscopic surgery did not require postoperative opioid analgesia. The average intensity of postoperative pain on the NRS for open cholecystectomy was slightly higher at 5.83, with a median of 8 (IQR 4–8), and an average of 28 mg equivalents of morphine was used compared to laparoscopic cholecystectomy, with an average NRS value of 4.76, a median of 5, and an IQR of 3–6, with 10 mg equivalents of morphine used. With the additional use of peripheral anesthesia, the mean NRS value was 3.5 [41].

An important element of pain management in the elderly and the underestimation of the incidence of postoperative pain is the difficulty associated with recognizing the presence of pain, often due to insufficient or difficult communication with the patient. In order to recognize and determine the intensity of postoperative pain, in addition to traditional numerical and verbal scales [Visual Analogue Scale (VAS), Verbal Rating Scale (VRS), Numeric Rating Scale (NRS)], it is recommended to use behavioral scales based on observations of nonverbal behaviors, vocalization, facial expression, changes in set activity, and a checklist of nonverbal pain indicators. The VRS is the preferred tool for patients with mild-to-moderate cognitive dysfunction. However, for patients with severe cognitive imbalance, behavioral scales such Doloplus−2 or Algoplus are necessary [5,6,42,43,44,45].

Current technological solutions exist for monitoring intraoperative analgesia with quantitative methods based on assessing autonomic system activity by monitoring capillary flow, chest impedance, and the skin’s condition [10,11,12,13,14]. An objective measurement of intraoperative pain based on the Surgery Pleth Index (SPI) suggests that maintaining an SPI above 60 predicts the occurrence of immediate postoperative pain rated as NRS > 5 with a sensitivity of 72% and a specificity of 88%, with a negative predictive value of 63 and a positive predictive value of 92% [46].

In order to reduce the use of opioid analgesia postoperatively, it is recommended to apply the principles of multimodal analgesia based on the use of the pharmacological synergism of analgesics and co-analgesics, combining the methods of regional and local anesthesia for surgical wounds. In addition, attention should be paid to the non-pharmacological aspects of supporting postoperative analgesia, with particular emphasis on preoperative education [47,48]. However, this does not mean categorically disqualifying opioids from use in elderly patients. Opioids are part of acute severe postoperative pain therapy. They are commonly administered via intravenous infusion or as a basic part of regional and local analgesia via epidural continuous infusion and postoperative wound infiltration. Patient-controlled analgesia (PCA) and patient-controlled epidural analgesia (PCEA) are crucial methods to avoid overdoses of opioids and provide rational therapy. The renal and cardiac changes inherent in aging influence the pharmacokinetics and pharmacodynamics of opioids. Additionally, cerebral changes increase nervous sensitivity to the sedative and psychotic effects of opioids.

The use of opioid drugs in the elderly should be modified due to the physiological changes associated with aging, especially slower metabolism and pharmacological detoxification and reduced intravascular volemia. Recommendations for opioid therapy in the elderly are described as “start slow and go slow”, prioritizing titration and observing the effects. A crucial suggestion is to use short-acting opioids and to reduce the doses. Doses of morphine should be reduced by 30–50% because of the active and high analgesic potency of morphine−6glucuronide as a main metabolite. Even the remifentanil dose should be reduced by 30% due to the decrease in the esterase level among the elderly, as well as the reduction in the volume of distribution by nearly 20% [6,24,25,26,27,28].

The opioid-limited anesthesia model is particularly applicable to the elderly patient population. In our study, we used an LOA pharmacological scheme for laparoscopic cholecystectomy based on limited administration of fentanyl with co-analgesics—lidocaine, ketamine, and magnesium sulfate—during anesthesia, with non-opioid analgesia (paracetamol and metamizole) postoperatively. However, the possibility of using the LOA technique for gastrointestinal, oncological, orthopedic, neurosurgical, and cardiac surgeries has also been described. The use of this method of analgesia in the postoperative period showed a reduction in the use of opioid drugs, as well as faster extubation and return of respiratory function. The lidocaine, ketamine, dexmedetomidine, and magnesium sulfate model was used for cardiac surgery anesthesia, and the study reported significantly lower opioid drug requirements expressed in morphine equivalents compared to the opioid anesthesia method, at 15 vs. 30 mg, respectively. In the 48 h postoperative period, there were no differences in the resting pain intensity or cough reflexes. In addition, there was a reduction in the number of incidents of atrial fibrillation, at 18 vs. 40, and a reduction in the use of non-invasive ventilation as respiratory support, at 25 vs. 48, respectively, (*p* < 0.05) [49]. Similar results for cardiac surgery anesthesia were presented for an OFA regimen using lidocaine, ketamine, and dexamethasone [22].

Toleska et al. [50] proposed Low-Opioid Anesthesia (LOA) for cancer surgery based on a single dose of 100 mcg of fentanyl intravenously and a bolus of 1 mg/kg of lidocaine, combined with epidural opioid administration. Opioid-free Anesthesia (OFA) consisted of the preoperative administration of 0.1 mg kg^−1^ of dexamethasone and 1 g of paracetamol, followed during the induction of anesthesia by lidocaine at 1 mg kg^−1^ and ketamine at 0.5 mg kg^−1^. In the maintenance phase, intravenous continuous infusion with lidocaine at 2 mg kg^−1^, ketamine 0.2 mg kg^−1^ h^−1^, and magnesium 15 mg kg^−1^ h^−1^ was administered. Both the LOA and OFA group showed a favorable analgesic trajectory, with a reduced incidence of vomiting compared to the OBA group. All methods had a negative slope trajectory. At 2 h postoperatively, the mean VAS values for the OBA, LOA, and OFA methods were 8.30, 8.10, and 4.90, respectively, and at the final examination, at 72 h, they were 3.75, 3.95, and 2.65, respectively. However, the mean VAS pain intensity score at 2, 12, 24, 36, and 72 h postoperatively was always significantly higher with the OBA method compared to the LOA and OFA methods. At 6 and 48 h, the mean VAS pain intensity score was highest with the LOA method, being 6.90 and 4.85, respectively, compared to the OBA method at 5.60 and 4.70, respectively, and the OFA method at 5.40 and 3.45, respectively [50].

The limitations of our study include the short time frame of clinical observation and the implementation of longer, less precise periods (6–12 h, 12–24 h). Despite this, we were able to collect data systematically. We did not use randomization in this study, and this causes additional limitations in interpretation. The scheme of analgesia assessment, or more precisely, the use of time intervals, is another important limitation of this study. For precise assessment, it is better to assess pain intensity at direct time points. In our study, the pharmacological scheme provided the dosing of drugs and anesthetics without taking into account each patient’s body weight. Body weight did not differ between groups, so we assumed our protocol to be correct. However, we are aware that such methodology is necessary in the pediatric population. Moreover, it is necessary to calculate the dose according to lean body weight in adult obese patients. However, there were a small number of patients, and the study group was monospecific. The collected data allowed for the analyses of short-term postoperative pain trajectories. Finally, two patients were excluded because of an incorrect analgesic regimen.

## 5. Conclusions

To conclude, this study reveals that OBA and non-opioid postoperative analgesia do not provide sufficient analgesia on the first postoperative day. This may be due to the belief and conviction that opioid drugs represent the greatest analgesic potency and that their use guarantees long postoperative comfort. However, additional observations from this study indicate that multidirectional analgesia involving a combination of minimal doses of opioid drugs and co-analgesics, as well as non-opioid analgesics, is more beneficial, especially in elderly patients.

Opioid analgesia in patients over 60 years of age presented a better effect in the immediate postoperative period. Non-opioid analgesia is indicated for patients over 60 years old in the later postoperative period. The model of combined minimal opioid anesthesia and non-opioid postoperative analgesia presents a favorable therapeutic effect for patients over 60 years old.

## Figures and Tables

**Table 1:** Patient characteristics in age categories. The median and minimum–maximum ranges are presented.

	LOA	OBA
***Category 1*** ***(≤40 years old)***	n = 4	n = 6
**Age**	35 (26–40)	35 (27–40)
**BMI**	25.5 (22–29)	23 (21–29)
**BSA**	1.8 (1.68–2.03)	1.81 (1.64–1.96)
**Duration of surgery (min)**	40 (35–50)	42 (38–55)
**Duration of anaesthesia (min)**	54 (45–65)	52 (45–70)
***Category 2*** ***(41–60 years old)***	n = 17	n = 19
**Age**	49 (41–60)	51 (41–60)
**BMI**	26 (18–34)	28 (19–41)
**BSA**	1.8 (1.56–2.07)	1.86 (1.54–2.14)
**Duration of surgery (min)**	43 (35–52)	45 (38–60)
**Duration of anaesthesia (min)**	55 (45–75)	56 (48–70)
***Category 3*** ***(>60 years old)***	n = 18	n = 12
**Age**	68 (63–85)	68 (63–81)
**BMI**	29.5 (20–38)	31 (24–38)
**BSA**	1.86 (1.61–2.21)	1.85 (1.63–2.35)
**Duration of surgery (min)**	45 (38–65)	50 (40–68)
**Duration of anaesthesia (min)**	55 (45–76)	60 (50–75)

## Data Availability

The data are available on special request to the authors.
